# CFD-derived radiomics from hemodynamic maps for quantitative assessment of left atrial flow in atrial fibrillation: a proof-of-concept study

**DOI:** 10.3389/fcvm.2026.1787926

**Published:** 2026-06-04

**Authors:** Lida Alinezhad, Paria Maleki, Camilla Cortesi, Corrado Tomasi, Alessandro Dal Monte, Matteo Falanga, Cristiana Corsi

**Affiliations:** 1 Department of Electrical, Electronic and Information Engineering, University of Bologna, Bologna, Italy; 2 Cardiology Department, Santa Maria delle Croci Hospital, AUSL della Romagna, Ravenna, Italy

**Keywords:** atrial fibrillation, computational fluid dynamics, hemodynamics, radiomics, texture analysis

## Abstract

**Background:**

Atrial fibrillation (AF) alters left atrial (LA) hemodynamics and increases thromboembolic risk. Computational fluid dynamics (CFD) can characterize complex flow patterns. However, extracting objective biomarkers from three-dimensional velocity fields remains challenging. This proof-of-concept study evaluates whether radiomics-based quantification of spatial texture patterns can provide reproducible LA flow biomarkers for AF classification.

**Methods:**

In this study thirty subjects (10 controls, and 20 AF patients) underwent contrast-enhanced cardiac computed tomography (CT). Five hemodynamic parameter maps (throughflow, wall-Parallelity Degree, normalized vorticity, local normalized helicity, and flow angle) across eight anatomical planes were generated from patient-specific CFD simulations. From 360 radiomics features, robust biomarkers were identified through nested leave-one-out cross-validation with three-stage feature selection: statistical filtering, effect-size thresholding, and stability assessment. Classification used the support vector machines with the radial basis function kernel. The model interpretability was evaluated using SHAP analysis.

**Results:**

Five robust features were identified. Four were Gray-Level Non-Uniformity features from throughflow regions, each achieving 100% selection frequency and bootstrap stability. The fifth was a Dependence Entropy feature from LAA helicity maps, with 80% selection frequency and 100% bootstrap stability. Effect sizes were large (*ε*^2^ = 0.46–0.60). The classifier achieved 93% accuracy, 95% sensitivity, 90% specificity, and an AUC of 0.92 (95% CI: 0.78–1.00). Permutation testing confirmed that performance significantly exceeded the chance level expected from random label assignment (*p* = 0.002). The features correlated significantly with LA enlargement (*ρ* = 0.45–0.64) and mitral regurgitation grade (*ρ* = 0.49–0.69), supporting biological plausibility.

**Conclusions:**

Radiomics analysis of CFD-derived hemodynamic maps can effectively quantify complex LA flow patterns and identify reproducible flow biomarkers in AF. The identified features demonstrated high stability and significant correlations with established markers of atrial remodeling. They also showed discriminative performance substantially exceeding that of clinical variables alone. External validation in larger multicenter cohorts is needed. This framework establishes the feasibility of objective hemodynamic phenotyping with potential applications in AF risk stratification.

## 1 Introduction

The atrial fibrillation (AF) is the most prevalent sustained cardiac arrhythmia, and is associated with a 4–5 fold increase in the risk of ischemic stroke (1). The underlying pathophysiology is closely linked to the left atrial (LA) hemodynamics: during AF, the loss of coordinated atrial contractions leads to a sluggish and disorganized LA blood flow, especially in the left atrial appendage (LAA) (2). This flow stagnation promotes thrombosis and subsequent embolization, thereby elevating the stroke risk in the AF patients (3). Intermittent AF carries a risk of blood clots comparable to that of persistent AF (1), indicating that even brief or self-terminating episodes can promote cardiac conditions conducive to clot formation. Recent evidences further demonstrate that AF-related flow abnormalities may persist beyond clinically apparent arrhythmia episodes; patients with a history of AF exhibit significantly reduced LA flow velocities and greater LA/LAA blood stasis compared to controls (4). Such alterations in atrial blood flow may increase thromboembolic risk even in the absence of ongoing fibrillation, highlighting the physiological and clinical significance of LA flow patterns in AF patients.

Assessing the LA blood flow in clinical practice presents significant challenges. Transthoracic echocardiography (TTE) is commonly used to evaluate transmitral inflow velocities (E/A waves) and overall diastolic function. Transesophageal echocardiography (TEE) remains the standard for investigating LAA flow and detecting stasis such as spontaneous echo contrast (“smoke”), or thrombi (5). Doppler TEE enables measurement of LAA emptying velocities to stratify stroke risk. TEE is semi-invasive, frequently necessitates sedation, and provides only limited insight into intra-atrial flow dynamics. Conventional echocardiography primarily yields one-dimensional velocity measurements along the ultrasound beam at a few sampled sites, such as the mitral valve and pulmonary veins, and therefore fails to capture the complex 3D flow field within the LA (6). This limitation hinders the comprehensive characterization of abnormal flow patterns in AF using ultrasound alone.

Four-dimensional flow cardiac MRI (4D flow CMR) enables volumetric, time-resolved measurement of blood velocities in three directions. Enabling precise quantification of complex LA flow phenomena, such as vortices and recirculation throughout the cardiac cycle (7). 4D flow studies show patients with paroxysmal AF have notably altered LA hemodynamics, including lower peak velocities, reduced kinetic energy, and increased stasis even during the sinus rhythm (8). However, 4D flow MRI has several limitations that restrict routine clinical application for LA flow assessment. The scans are time-consuming, spatial resolution (approximately 2.5–3 mm) is relatively coarse, and sensitivity to very low velocities is limited, which impeding assessment of sluggish flow in the LAA (9, 10). These challenges highlight the need for more robust and accessible methods to characterize LA flow.

Cardiac computed tomography (CT) offers an alternative approach, providing high-resolution anatomical imaging with progressively reduced radiation exposure (11). Although CT does not directly measure blood velocity, it reveals cardiac chambers in detail throughout the cardiac cycle when contrast is administered. By tracking chamber boundary motion across sequential 3D frames, it is possible to compute volume flow rates, such as across the mitral valve or the LAA orifice, by calculating the time derivative of chamber volume change (11). CT-based computational fluid dynamics (CT-CFD) simulation has emerged as a powerful approach to characterize LA hemodynamics in AF patients, yielding detailed maps of intra-atrial flow velocity, vorticity, wall shear stress, and blood residence time (12, 13). Recent CT-CFD studies have demonstrated that AF-related atrial remodeling leads to diminish LA flow velocities, elevated blood stasis, and reduced wall shear stress in the LAA compared to normal sinus rhythm (14–17). These hemodynamic changes correlate with increased LAA thrombosis risk, underscoring the clinical relevance of CT-CFD for assessing patient-specific blood stasis and thromboembolic risk in AF (12, 18). A notable recent CFD study demonstrated elevated atrial blood stasis in paroxysmal AF patients even during sinus rhythm, providing mechanistic support for the persistent stroke risk observed in this population (19).

Radiomics has been proposed to improve these simulations by extracting quantitative features from simulated flow fields to characterize complex flow patterns. The power of radiomics lies in its ability to detect subtle textural or spatial patterns that elude visual inspection, thereby enabling identification of imaging “signatures” of the disease phenotypes. Notably, radiomics analysis of cardiac CT has demonstrated value in differentiating LAA thrombus from circulatory stasis, with texture features achieving higher discriminative performance than conventional attenuation-based metrics (20, 21). Conventional CFD analysis typically reports scalar summary statistics, such as mean velocity or the percentage of LA volume with stasis, that reduce complex three-dimensional flow fields to single values. However, two patients with identical mean velocities may exhibit fundamentally different spatial patterns: one with organized, laminar flow and the other with fragmented, chaotic flow throughout the chamber. Radiomics texture features address this limitation by quantifying how spatially similar velocities are distributed, thereby capturing flow pattern complexity that scalar metrics inherently discard.

Huellebrand et al. applied a radiomics framework to 4D flow MRI data of the aorta, demonstrating that radiomics features derived from hemodynamic parameter maps (throughflow, vorticity, helicity) could quantitatively characterize complex flow profiles with high reproducibility (22). Their study identified features that successfully differentiated flow patterns by patient age, sex, and aortic pathology, establishing proof of principle that complex hemodynamic phenotypes can be objectively described using texture-based image features.

The present study extends this radiomics framework to CFD-derived hemodynamics maps of the left atrium in AF patients. To the best of our knowledge, this effort represents the first application of radiomics to LA flow characterization. It was hypothesized that texture features extracted from hemodynamic parameter maps can objectively quantify the spatial disorganization of LA blood flow characteristic of AF. To be more specific, this proof-of-concept study aims to: 1) identify robust radiomics biomarkers that characterize LA flow in AF with high stability across validation frameworks, and 2) evaluate diagnostic performance compared to clinical parameters alone and assess biological plausibility through correlation with established markers of atrial remodeling.

## 2 Materials and methods

In this section, the study cohort and the methodology is described.

### 2.1 Study population and data acquisitions

This study analyzed the data from the FATA (Fluid-dynamics in the Left Atrium in Atrial Fibrillation Patients and Controls for Thrombogenic Risk Analysis) study, conducted at “S. Maria delle Croci” Hospital, located at Ravenna, Italy. The study was approved by the Local Ethics Committee of Romagna (C.E.R.O.M., approval no. 1276/2019 I.5/6, 13 February 2019) (17). The cohort involved 30 subjects: 10 controls in sinus rhythm and 20 patients with AF, classified according to clinical criteria established in Ref. (23). Contrast-enhanced cardiac CT images were acquired using a Philips Brilliance 64-slice scanner, with all subjects in sinus rhythm. Each scan consisted of approximately 170 axial slices, with an in-plane resolution of 0.4 mm and a slice thickness of 1.0 mm. All images were exported in DICOM format for subsequent segmentation and computational flow simulation.

Clinical and baseline demographic data were collected for all participants, as summarized in Table 1. Clinical data included age, gender, body surface area (BSA), left atrial enlargement grade (0–3 scale by echocardiography), mitral regurgitation grade (0–4 scale), and left ventricular ejection fraction, with 100% completeness for age/gender and 96.7% (29/30) for other variables. In this population, 80% of participants were men. This male predominance matches what is commonly observed in AF populations (24).

**Table 1:** Baseline demographics and clinical characteristics of the study population.

Characteristics	Control *(n = 10)*	Paroxysmal AF^§^ (*n = 10*)	Persistent AF (*n = 10*)	*P-value*
Demographics				
Age, years	61.1 ± 10.2	62.1 ± 5.5	66.5 ± 9.7	0.357
Male, *n* (%)	8 (80.0)	8 (80.0)	8 (80.0)	1.000
BSA, m^2^	1.9 ± 0.2	2.0 ± 0.1	2.1 ± 0.2	0.097
Echocardiographic findings				
LVEF	0.60 ± 0.04	0.58 ± 0.06	0.59 ± 0.05	0.587
LA enlargement grade^†^				0.011*
None	8 (80.0)	3 (33.3)	2 (20.0)	
Mild	2 (20.0)	2 (22.2)	3 (30.0)	
Moderate	0 (0.0)	3 (33.3)	3 (30.0)	
Severe	0 (0.0)	1 (11.1)	2 (20.0)	
Mitral regurgitation grade^‡^				0.009*
None	9 (90.0)	3 (33.3)	3 (30.0)	
Mild	1 (10.0)	2 (22.2)	2 (20.0)	
Mild-moderate	0 (0.0)	3 (33.3)	4 (40.0)	
Moderate-severe	0 (0.0)	1 (11.1)	1 (10.0)	

AF, atrial fibrillation; BSA, body surface area; LA, left atrial; LVEF, left ventricular ejection fraction.

Values are presented as mean ± SD or *n* (%).

† LA enlargement graded as: 0 = none, 1 = mild, 2 = moderate, 3 = severe.

‡ Mitral regurgitation graded as: 0 = none, 1 = mild, 2 = mild-moderate, 3 = moderate-severe, 4 = severe.

§ *n* = 9; one patient with missing echocardiographic data.

* Statistically significant (*P* < 0.05); *P* values derived from one-way ANOVA for continuous variables. For ordinal variables (LA enlargement, MR grade), *P* values correspond to the continuous grading scale; Fisher's exact test was used for categorical comparisons due to small cell sizes.

### 2.2 Computational fluid dynamics and hemodynamic parameter extraction

Patient-specific 3D LA geometries were reconstructed from CT images and used as input for computational fluid dynamics (CFD) simulations performed in *LifeX* (17, 25). The complete CFD specification, including boundary conditions, mesh parameters, wall kinematics, solver settings, and flow regime assumptions, is provided in Supplementary Material A.

The time point corresponding to peak left atrial (LA) volume, which aligns with ventricular end-systole, was selected for radiomics extraction. At this phase, the atrium has accumulated blood during systolic filling, and intra-atrial flow patterns, including regions of stagnation, recirculation, and disorganized motion, are most fully developed. This marks the phase when atrial fibrillation (AF)-related hemodynamic alterations are expected to be most pronounced. Furthermore, peak LA volume provides the largest spatial extent for texture-feature computation, ensuring sufficient voxel counts for reliable higher-order statistical analysis. In addition, this methodology aligns with the approach of Huellebrand et al. (22), who also selected a single peak-systolic phase for radiomics-based flow classification, despite access to temporally resolved four-dimensional (4D) flow MRI data.

**Figure 1:**
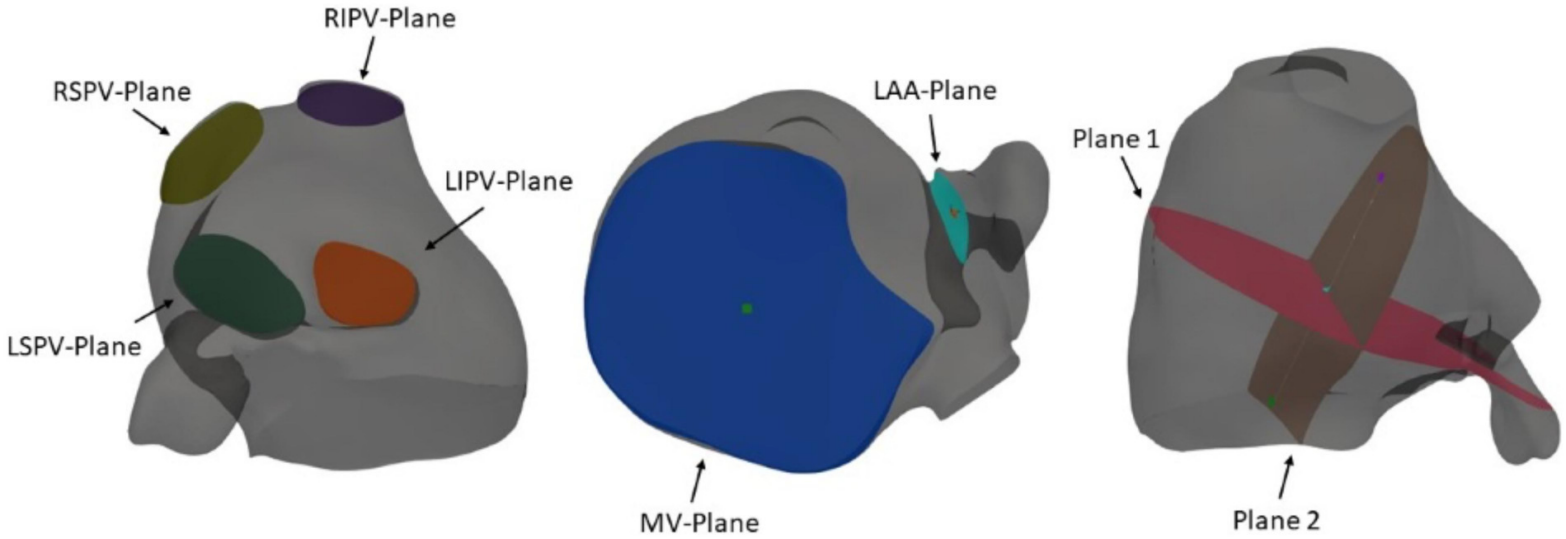
Eight planes were defined at critical anatomical location for capturing the relevant hemodynamic parameters.

Eight anatomical planes were defined within the LA geometry based on the key anatomical landmarks: mitral valve (MV), four pulmonary vein ostia including left superior pulmonary vein (LSPV), left inferior pulmonary vein (LIPV), right superior pulmonary vein (RSPV), and right inferior pulmonary vein (RIPV), left atrial appendage (LAA), and two internal reference planes (Plane 1 and Plane 2) positioned orthogonal and parallel to the atrial centerline, respectively. These planes are shown in Figure 1.

Five hemodynamic parameter maps were computed for each plane using ParaView which are Throughflow, Wall-Parallelity Degree (WPD: flow alignment with local wall surface), Normalized Vorticity component (Ω_*n*_: rotational flow intensity), Local Normalized Helicity (LNH: helical flow organization), and Flow Angle (22). These parameters quantify distinct aspects of blood flow organization, including through-plane transport, directional alignment, rotational motion, and helical flow characteristics (26). A detailed description of the hemodynamic parameters is reported in Supplementary Material B. Parameter values were rescaled to standardized ranges following established conventions: symmetric ranges [−2,048, 2,047] for throughflow, LNH, and $\Omega_{n}$ and [0, 4,096] for WPD and flow Angle (22). Parameters normalization is detailed in Supplementary Material C. This standardization ensures consistent discretization for subsequent radiomics feature extraction.

### 2.3 Radiomics feature extraction

Hemodynamic parameter maps were converted to NRRD format and resampled to isotropic 1 × 1 mm^2^ in-plane resolution using B-spline interpolation to ensure spatial standardization across subjects (27). Binary masks were generated to isolate flow-relevant regions, in which voxels representing non-zero parameter values were assigned to a label of 1. Radiomics features were extracted using PyRadiomics^1^ (version 3.0.1) in accordance with the Image Biomarker Standardization Initiative (IBSI) guidelines (28, 29). Intensity discretization employed a fixed bin width of 25 gray levels for texture feature computation, consistent with recommendations for continuous-valued medical imaging data. For each subject, 360 features were extracted across the eight anatomical planes and five hemodynamic parameters, comprising first-order statistics, 2D shape descriptors, and texture features from Gray Level Co-occurrence Matrix (GLCM), Gray Level Run Length Matrix (GLRLM), Gray Level Size Zone Matrix (GLSZM), Gray Level Dependence Matrix (GLDM), and Neighboring Gray Tone Difference Matrix (NGTDM). The complete preprocessing pipeline, normalization specifications, and PyRadiomics configuration are provided in the Supplementary Material D.

### 2.4 Feature selection and robustness assessment

All AF patients (paroxysmal and persistent) were combined into a single AF group for binary classification against controls, given the limited sample size (*n* = 10 per subgroup) for subtype analysis. A nested leave-one-out cross-validation (LOOCV) framework was implemented to ensure unbiased performance estimation and to maximize data utilization given the small sample size (30, 31). Within each LOOCV fold, feature selection was performed exclusively on the training set (*n* = 29) using a three-stage pipeline as follows:

1. **Univariate filtering:** Kruskal–Wallis *H*-test assessed discriminative power, given the small sample size and non-normal distribution of radiomics features (32).
2. **Multiple testing correction:** Benjamini–Hochberg false discovery rate (FDR) correction at *α* = 0.10 controlled false discoveries while maintaining sensitivity for biomarker discovery (33).
3. **Effect size filtering:** Epsilon-squared (*ε*^2^) ≥ 0.14, calculated as (*H* − *k* + 1)/(*n* − *k*) where *H* is the Kruskal–Wallis statistic, *k* is the number of groups, and *n* is the sample size, ensured practical significance beyond statistical significance (34). This threshold corresponds to a large effect size for non-parametric tests, ensuring retained features explain substantial between-group variance.

Features passing all criteria were ranked by effect size. A maximum of five features was selected per fold, maintaining a sample-to-feature ratio of 6:1. This represents a pragmatic compromise between the conservative *one in ten* rule (35), and the practical constraints of small-sample radiomics, where *n*/5 features is considered acceptable when combined with rigorous cross-validation.

Feature stability was evaluated through multiple independent frameworks to identify reproducible biomarkers (37). LOOCV selection frequency was calculated across all 30 folds. Features selected in ≥70% of folds were considered reproducible. This threshold follows Meinshausen and Bühlmann's stability selection framework (38), which recommends thresholds between 50% and 90%; we selected 70% as a stringent criterion ensuring high reproducibility while avoiding excessive conservatism (38).

Bootstrap stability analysis employed stratified sampling with 1,000 iterations and 70% sampling per group. Within each bootstrap iteration, features were selected if their Kruskal–Wallis *p*-value fell below *α* = 0.10. FDR correction was not applied per iteration to avoid excessive conservatism in subsampled data. Features achieving ≥70% selection frequency across iterations were classified as highly stable. The 70% threshold represents the theoretical lower bound for controlling the false discovery rate in stability selection (38). A composite robustness scores integrated LOOCV frequency with 40% weight, bootstrap stability with 40% weight, and normalized effect size with 20% weight. The final feature assessment on the full sample employed FDR correction, *q* < 0.10, to control false discoveries.

The features simultaneously meeting all criteria (LOOCV frequency ≥70%, bootstrap stability ≥70%, *ε*^2^ ≥ 0.14, FDR *q* < 0.10) were classified as highly robust biomarkers. This process identified eight highly robust features; the top five features ranked by composite robustness score were used for classification to maintain the 6:1 sample-to-feature ratio.

### 2.5 Radiomics-clinical correlation analysis

Associations between the five robust radiomics features and clinical parameters (age, BSA, LA enlargement, MR grade, and LV EF) were assessed using Spearman's rank correlation coefficient (39). Correlations were computed across the full cohort (*n* = 30) to identify relationships between hemodynamic texture features and clinical markers of atrial remodeling. Multiple testing was controlled using FDR correction; correlations with *q* < 0.10 were considered statistically significant (33).

### 2.6 Machine learning classification

Support vector machine (SVM) was selected for classification, given its effectiveness in modeling non-linear decision boundaries in small-sample radiomics datasets (40, 41). All SVM hyperparameters were fixed *a priori* (C = 1.0, gamma = “scale”, RBF kernel) without cross-validated tuning, providing a conservative performance estimate and avoiding overfitting risk inherent in nested hyperparameter optimization with small sample sizes. The class imbalance of 10 controls vs. 20 AF was addressed using balanced class weights, which inversely weight classes proportional to their frequency (42). The radiomics model utilized the five robust features identified through stability selection. The complete machine learning pipeline parameters are reported in Supplementary Material E.

#### 2.6.1 Model performance evaluation

Model performance was evaluated using the nested LOOCV framework described in Section 2.4. Feature standardization (*z*-score normalization) was performed within each fold using only training data statistics to prevent data leakage (43, 44). Performance metrics included the area under the receiver operating characteristic curve (AUC-ROC), sensitivity, specificity, and balanced accuracy.

#### 2.6.2 Model interpretability

To clarify which radiomics features drive the classifier's decisions, Shapley Additive Explanations (SHAP) analysis on the final SVM model was performed. SHAP values quantify each feature's contribution to individual predictions and provide model-agnostic interpretability based on game-theoretic principles (45, 46). The model was trained on all 30 patients using the five robust features identified through stability selection. SHAP values were computed using Kernel SHAP with all samples as background, returning AF class probabilities. Feature importance was assessed via mean absolute SHAP values, while directional impact was evaluated through group-specific means.

Given the small cohort (*n* = 30) and high-dimensional feature space (360 features), particular attention was paid to mitigate the overfitting. The initial sample-to-feature ratio (*n*/*p* ≈ 0.08) represents a classic *curse of dimensionality* scenario. Established guidelines recommend 10–15 observations per variable for reproducible estimation (35, 36, 47), with radiomics studies suggesting retaining only 5%–10% of features in small datasets and final models containing fewer than 10 features. Our nested cross-validation pipeline ultimately selected five highly robust features (final *n*/*p* = 6:1), balancing biological interpretability with overfitting mitigation. To confirm that classifier performance exceeded chance levels, permutation testing was performed with 1,000 iterations of randomly shuffled class labels, repeating the complete nested LOOCV pipeline for each permutation. The empirical *p*-value was calculated as (*r* + 1)/(*n* + 1), where r is the number of permuted accuracies equal to or exceeding the observed accuracy, following the correction proposed by Phipson and Smyth to avoid zero *p*-values (48).

### 2.7 Software implementation

All analyses were conducted in Python (version 3.10) using open-source libraries: PyRadiomics 3.0.1 for IBSI-compliant feature extraction (29), scikit-learn 1.3 for machine learning (49), NumPy 1.26 and pandas 2.1 for data analysis (50, 51), SciPy 1.11 for statistical analysis (52), SHAP 0.43 for interpretability (53), Matplotlib 3.7, and Seaborn for visualization (54, 55).

## 3 Results

The results of this study are organized as follows: evaluation of model performance in classifying AF patients vs. controls using radiomics features derived from hemodynamic maps; identification of the most robust features within the study cohort; assessment of model interpretability; and analysis of the correlation between clinical and CFD-derived radiomics features.

### 3.1 Model performance

**Figure 2:**
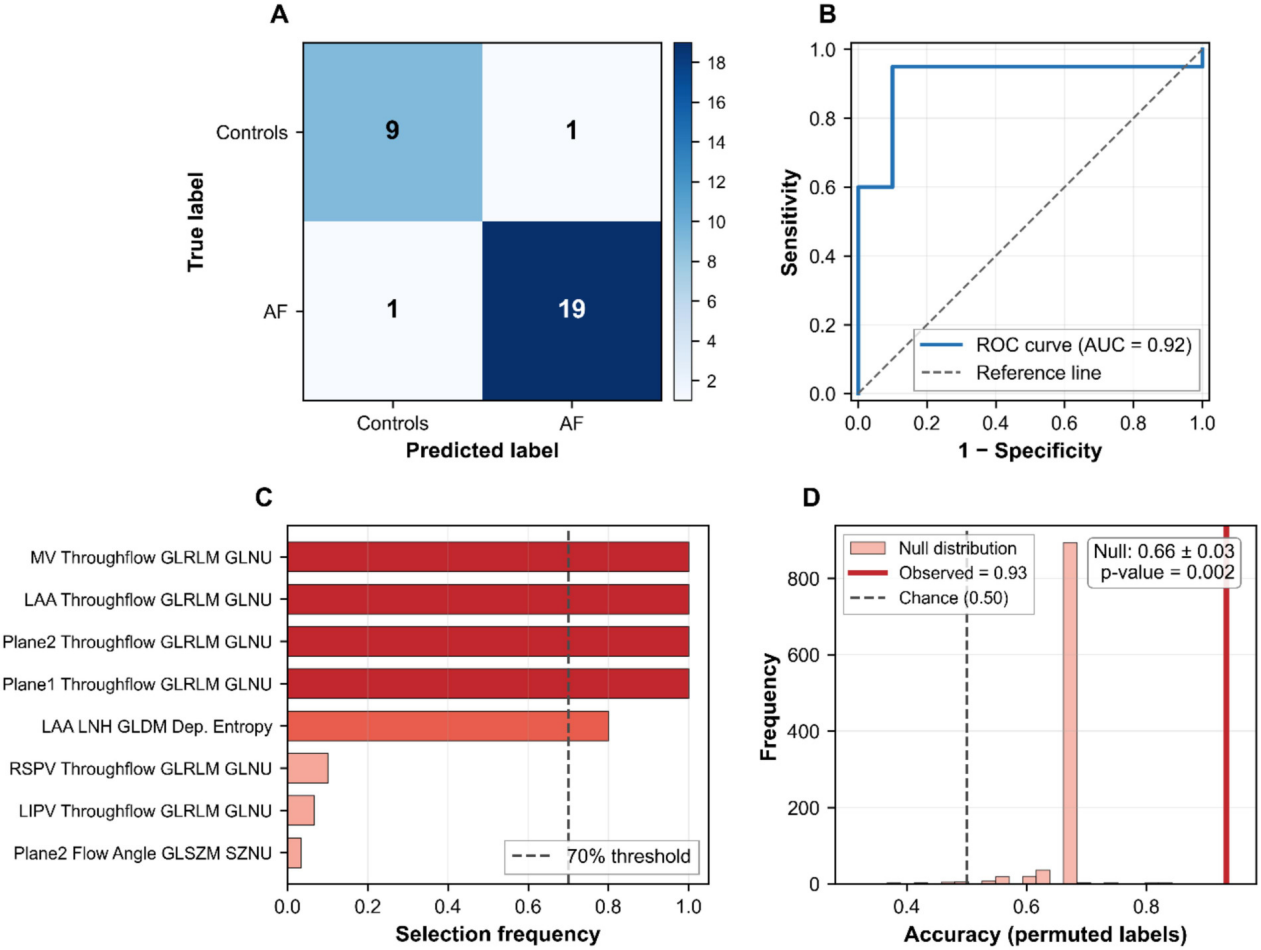
SVM classifier performance for AF detection: **(A)** represents confusion matrix for *n* = 30, **(B)** represents ROC curve (AUC = 0.92), **(C)** represents feature selection frequency across LOOCV folds with dashed line indicating 70% stability threshold, and **(D)** represents the permutation test.

The SVM classifier effectively differentiated blood flow patterns between the two populations. The confusion matrix presented in Figure 2A shows that 19 of 20 AF patients were correctly classified, while 9 of 10 controls were correctly identified, yielding one false negative and one false positive. The receiver operating characteristic curve in Figure 2B reveals robust discriminative performance with an area under the curve of 0.92, with the dashed diagonal representing random classification. Bootstrap resampling (1,000 iterations) yielded 95% confidence intervals: accuracy 93.3% (83.3%–100.0%), sensitivity 95.0% (84.0%–100.0%), specificity 90.0% (66.7%–100.0%), and AUC-ROC: 0.92 (0.78–1.00).

Feature stability analysis in Figure 2C displays the eight radiomics features ranked by selection frequency across leave-one-out cross-validation (LOOCV) folds. All features surpassed the 70% stability threshold, as indicated by the dashed line, confirming high reproducibility.

Permutation testing with 1,000 iterations, as shown in Figure 2D confirmed that classifier performance significantly exceeded chance level (observed accuracy = 93.3%, null distribution mean = 65.9% ± 3.5%, *p* = 0.002).

### 3.2 Feature selection and robustness

The nested feature selection pipeline identified five highly robust radiomics features that consistently characterized blood flow in AF patients and controls, as shown in Table 2. Four features measuring Gray-Level Non-Uniformity in throughflow regions demonstrated promising stability, each achieving LOOCV selection frequency of 1.000 and bootstrap stability of 1.000.

**Table 2:** List of highly robust radiomics features for AF classification.

Feature	LOOCV frequency	Bootstrap stability	Effect Size (*ε*^2^)	FDR q-value	Final score
*Plane 1 Throughflow GLRLM GLNU*	1.000	1.000	0.601	8.63 × 10^−5^	1.000
*Plane 2 Throughflow GLRLM GLNU*	1.000	1.000	0.562	8.63 × 10^−5^	0.987
*LAA Throughflow GLRLM GLNU*	1.000	1.000	0.549	8.63 × 10^−5^	0.983
*MV Throughflow GLRLM GLNU*	1.000	1.000	0.524	9.39 × 10^−5^	0.974
*LAA LNH GLDM Dependence Entropy*	0.800	1.000	0.464	1.84 × 10^−4^	0.874

AF, atrial fibrillation; FDR, false discovery rate; GLDM, gray-level dependence matrix; GLRLM, gray-level run-length matrix; LAA, left atrial appendage; LNH, local normalized helicity; LOOCV, leave-one-out cross-validation; MV, mitral valve.

The top-ranked feature, *Plane1_Throughflow_glrlm_GrayLevelNonUniformity* (Gray-Level Non-Uniformity feature of the GLRLM computed from the Throughflow map on Plane1) achieved a final robustness score of 1.000 and demonstrated the largest effect size (*ε*^2^ = 0.601, *q* < 0.0001). This feature quantifies the spatial heterogeneity of velocity patterns in the primary throughflow region connecting the pulmonary veins to the mitral valve. Three additional throughflow-region features on the LAA ostium, the plane inside the LA chamber and orthogonal to the centerline and the mitral valve plane (*Plane2_Throughflow*, *LAA_Throughflow*, and *MV_Throughflow*) measuring the same textural property showed similarly high robustness scores (0.987, 0.983, and 0.974, respectively) with large effect sizes (*ε*^2^ = 0.524–0.562, all *q* < 0.0001), suggesting that disrupted flow organization in the throughflow pathway is a consistent and spatially distributed marker of AF patients.

The fifth robust feature, *LAA_LNH_gldm_DependenceEntropy* (Dependence Entropy feature of the GLDM computed from the LNH map on the LAA ostium) with LOOCV frequency: 0.8, bootstrap stability: 1, final score: 0.874, characterizes the complexity of low-normalized-helicity regions in the left atrial appendage. This feature captured a moderate effect size (*ε*^2^ = 0.464, *q* < 0.001), reflecting the altered vortical flow patterns and reduced helical organization within the LAA that accompany AF patients.

### 3.3 Model interpretability

**Figure 3:**
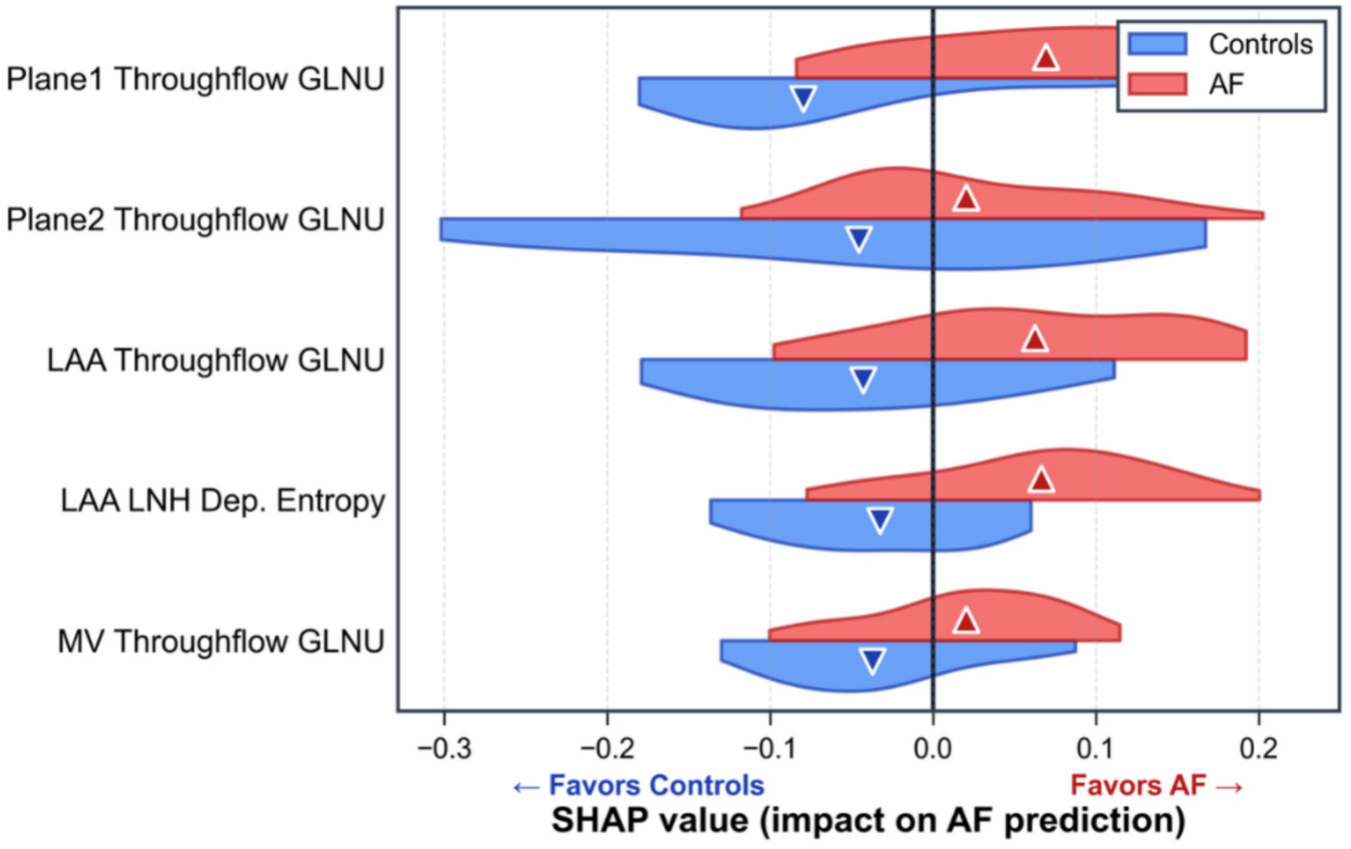
SHAP analysis of radiomics feature contributions to AF classification. Split violin plot comparing SHAP value distributions between Controls (blue) and AF patients (red) for all robust features.

SHAP analysis revealed consistent directionality across all five robust features, shown in Figure 3. AF patients exhibited positive SHAP values in favor of AF classification, and Controls showed negative values favoring Controls classification. Throughflow GLNU on Plane1 demonstrated the largest group separation, followed by Throughflow GLNU on Plane2 and Throughflow GLNU at the ostium of the LAA. These throughflow-derived features showed clear distributional shifts between groups, indicating that elevated Gray-Level Non-Uniformity values consistently drive AF prediction. LAA-derived features, such as LAA Throughflow GLNU and LAA LNH Dependence Entropy, further reinforced the established role of the appendage in AF pathophysiology, with AF patients showing distinctly higher SHAP contributions compared to Controls.

### 3.4 Radiomics-clinical integration

**Figure 4:**
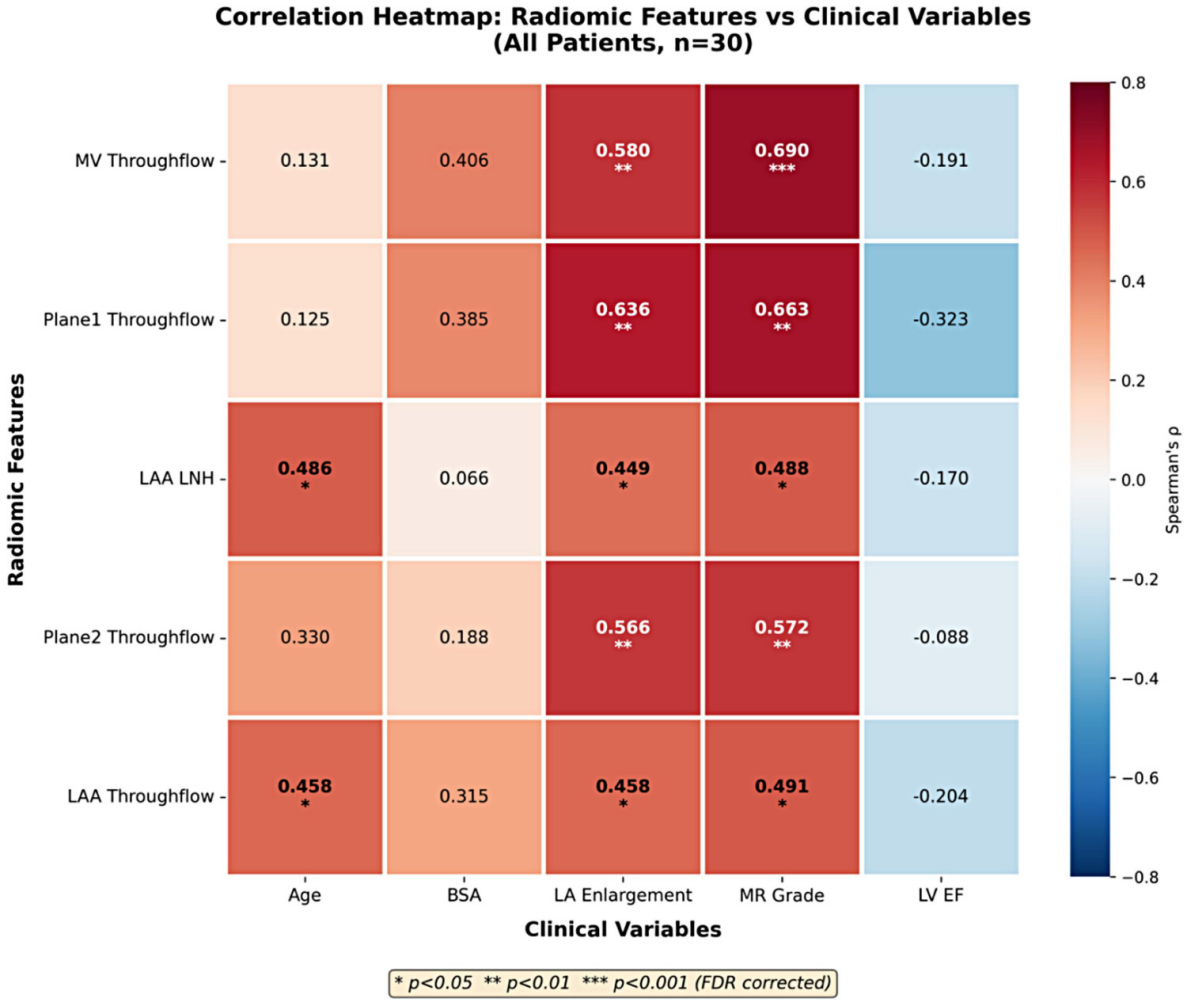
Robust radiomics-clinical features correlation matrix.

To examine the relationship between hemodynamic-derived radiomics features and clinical parameters, Spearman correlation analysis was performed, as shown in Figure 4. All five radiomics features demonstrated significant positive correlations with MR grade (*ρ* = 0.488–0.690, FDR-corrected *p* < 0.01), with Throughflow GLNU at MV exhibiting the strongest association (*ρ* = 0.690, *p* < 0.001). All five features also correlated significantly with LA enlargement (*ρ* = 0.449–0.636, *p* < 0.05), suggesting that these texture features capture flow patterns associated with both valvular dysfunction and atrial remodeling. LAA-derived features showed age-related associations (*ρ* = 0.458–0.486, *p* < 0.05), while no significant correlations were observed with LV EF (all *p* > 0.05), consistent with preserved systolic function in this cohort. These findings support the physiological relevance of throughflow-derived radiomics features as imaging biomarkers of AF related hemodynamic alterations.

**Figure 5:**
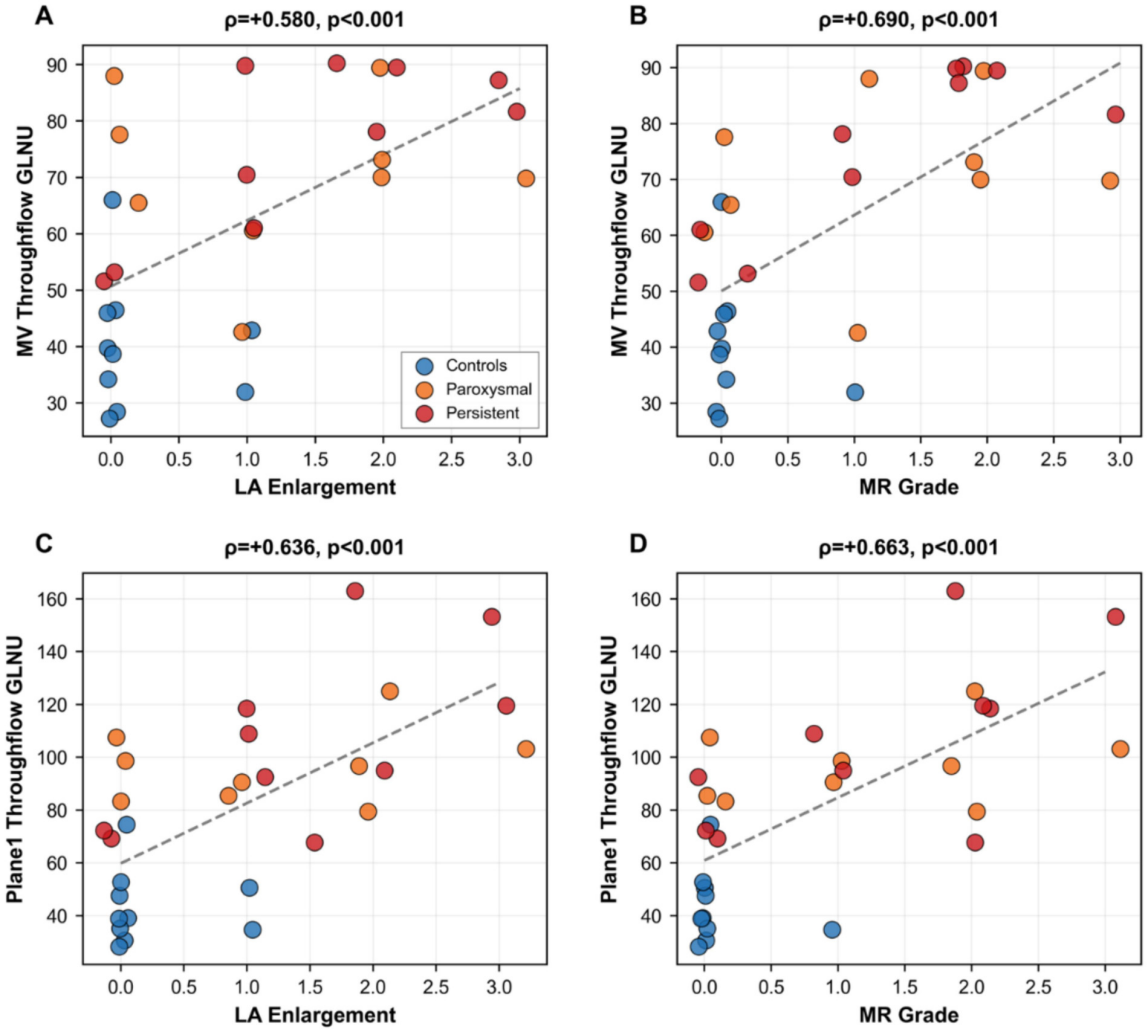
Correlation between top radiomics features and clinical parameters. **(A)** presents the correlation between Throughflow GLNU at MV and LA enlargement (*ρ* = +0.580, *p* < 0.001), **(B)** presents the correlation between Throughflow GLNU at MV and MR grade (*ρ* = +0.690, *p* < 0.001), **(C)** presents the correlation between Throughflow GLNU on Plane 1 and LA enlargement (*ρ* = +0.636, *p* < 0.001), and **(D)** presents the correlation between Throughflow GLNU on Plane 1 and MR grade (*ρ* = +0.663, *p* < 0.001).

As it can be seen in Figure 5, the two highest ranking radiomics features demonstrated significant correlations with established clinical markers of AF severity. In Figure 5A, Throughflow GLNU at MV showed strong positive correlations with both LA enlargement (*ρ* = 0.580, *p* < 0.001) and MR grade (*ρ* = 0.690, *p* < 0.001), see Figure 5B. Similarly, Throughflow GLNU on Plane1 correlated significantly with LA enlargement (*ρ* = 0.636, *p* < 0.001), shown in Figure 5C and MR grade (*ρ* = 0.663, *p* < 0.001) shown in Figure 5D. Visual inspection revealed consistent group stratification across all panels, with Controls (blue) clustering at lower feature values and clinical severity scores, paroxysmal AF (orange) at intermediate levels, and persistent AF (red) exhibiting the highest values for both radiomics features and clinical parameters. These associations suggest that throughflow-derived texture features capture hemodynamic alterations that parallel structural and functional remodeling in AF.

To determine whether the radiomics signal reflects AF-specific hemodynamics or related structural remodeling, an incremental analysis was conducted. The clinical-only model, which included age, gender, body surface area, LV EF, LA enlargement grade, and MR grade, demonstrated near-chance performance (AUC = 0.57). In contrast, the radiomics model achieved a significantly higher AUC of 0.92. Incorporating clinical variables into the radiomics model did not yield further improvement (AUC = 0.92), indicating that the hemodynamic texture features capture spatial flow complexity that surpasses the discriminative capacity of clinical surrogates for atrial remodeling. Further details are reported in Supplementary Material F.

## 4 Discussion

This study demonstrates that radiomics analysis of CFD-derived hemodynamic maps can quantify left atrial flow patterns in AF. The classifier achieved 93% accuracy (AUC = 0.92) in distinguishing blood flow features in AF patients from controls. Five robust biomarkers were identified. Four measured spatial heterogeneity of throughflow patterns (Gray-Level Non-Uniformity) and one captured as vortical flow complexity (Dependence Entropy). These features showed significant correlations with established clinical markers, including LA enlargement (*ρ* = 0.45–0.64) and MR grade (*ρ* = 0.49–0.69).

### 4.1 Novel contributions

The principal contribution of this work is applying radiomics methodology to CFD-derived hemodynamic fields. Previous studies have mainly relied on scalar summary statistics such as mean velocity, peak velocity, or volumetric stasis thresholds to characterize atrial flow. Although informative, these approaches compress complex spatiotemporal patterns into single values. This may discard diagnostic relevant information. The present radiomics framework addresses this limitation by computing second-order textural features (GLCM, GLRLM, GLSZM, GLDM, and NGTDM). These features encode spatial relationships between adjacent flow regions. They preserve information about flow pattern complexity that scalar metrics cannot capture.

The predominance of GLNU features from throughflow regions merits particular attention. This GLRLM-derived metric quantifies spatial heterogeneity of consecutive voxels with similar intensity values (56). Elevated GLNU values indicate fragmented flow patterns where regions of similar velocity are spatially discontinuous. GLNU features were consistently selected across multiple anatomical planes (MV, LAA, Plane1, and Plane2). This suggests that flow fragmentation is a spatially distributed characteristic of AF hemodynamics. This outcome is consistent with the loss of coordinated atrial contraction that produces multidirectional flow throughout the chamber (57).

The inclusion of Dependence Entropy derived from the GLDM of the LNH map at the LAA ostium (*LAA_LNH_gldm_DependenceEntropy*) provides complementary information about vortical flow complexity. Dependence Entropy quantifies randomness in the distribution of connected voxel neighborhoods (56). It captures the unpredictability of vortex organization in regions of low normalized helicity. The combination of run-length texture and dependence-based entropy features indicates that AF produces both fragmented velocity patterns and disorganized rotational structure.

### 4.2 Clinical correlations

The correlations between radiomics features and clinical parameters support biological plausibility. GLNU of the Throughflow map computed on Plane1 correlated significantly with LA enlargement (*ρ* = 0.636, *p* < 0.001) and MR grade (*ρ* = 0.663, *p* < 0.001). This is consistent with the known relationship between atrial structural remodeling, valvular dysfunction, and hemodynamic impairment (58).

GLNU of the Throughflow map computed on the MV plane demonstrated the strongest correlation with MR grade (*ρ* = 0.690, *p* < 0.001). This suggests that flow texture immediately proximal to the mitral valve is particularly sensitive to mitral regurgitation pathology. From mechanistic perspective, mitral regurgitation creates retrograde jets that disrupt organized transmitral inflow.

Age-related associations were observed in LAA features (LAA LNH Dependence Entropy: *ρ* = 0.486; LAA Throughflow GLNU: *ρ* = 0.458). These findings are consistent with progressive deterioration of atrial function with aging (59, 60). The absence of correlation with LV ejection fraction (all *p* > 0.05) reflects the preserved systolic function in our cohort. This indicates that these radiomics signatures capture atrial-specific hemodynamic alterations rather than general cardiac dysfunction.

### 4.3 Model interpretability

SHAP analysis revealed consistent directionality across all five robust features. AF patients exhibited positive SHAP values, favoring AF classification. Controls showed negative values, favoring Controls classification. Throughflow-derived features from the primary anatomical planes (Plane 1, Plane 2) demonstrated the largest group separation. LAA-derived features (Throughflow GLNU and LNH Dependence Entropy on the LAA ostium) showed similar patterns, reinforcing the established role of the appendage in AF pathophysiology. The clear separation between groups across all features supports the biological relevance and interpretability of the classifier.

### 4.4 Biological interpretation

The spatial distribution of robust features inside the LA chamber (Plane 1 and Plane 2), at the LAA ostium and at the MV suggests that AF produces chamber-wide alterations in flow topology. This finding challenges models focusing exclusively on LAA stasis as the primary thrombogenic mechanism. While LAA stagnation remains important, our results indicate that flow disruption extends throughout the LA cavity.

The selection of the Dependence Entropy Feature computed from LNH map at the LAA ostium as the sole helicity-based feature is noteworthy. This feature may serve as a marker of thrombogenic potential. It identifies appendages with disorganized, stagnant flow patterns that may be at elevated risk for clot formation. Future prospective studies are needed to validate this hypothesis.

### 4.5 Limitations

Several limitations of this study warrant consideration. First, the cohort size was limited to 30 subjects. Although the nested LOOCV framework and permutation testing were specifically chosen to maximize statistical validity under these constraints, the sample size restricts both statistical power and generalizability. External validation in larger, multi-center cohorts is required. Second, although the CFD simulations employed moving-boundary conditions across the cardiac cycle, the hemodynamic parameter maps were extracted at a single time point (peak LA volume at ventricular end-systole) for radiomics analysis. This phase captures the largest intracardiac blood volume and ensures geometric consistency across subjects. However, flow patterns change throughout the cardiac cycle, and features extracted at alternative phases may provide complementary information. The single-phase extraction approach aligns with Huellebrand et al. (22), who also selected a peak-systolic timeframe despite access to temporally resolved 4D flow MRI data. Previous studies have demonstrated that AF-related hemodynamic alterations persist throughout the cardiac cycle (1, 6), indicating that differences observed at peak LA volume likely represent chronic flow disruption rather than phase-specific effects. Nonetheless, formal assessment of temporal robustness across multiple cardiac phases remains an important direction for future research. Third, no distinction was made between paroxysmal and persistent AF subtypes. Although clinical correlations suggested group separation, future adequately powered studies should address subtype differentiation. Fourth, the dependence of the CFD model on boundary conditions and on grid and time-step independence has not been tested for key hemodynamic quantities and warrants future investigation. Fifth, this study employed a case-control design without hard clinical endpoints. Prognostic utility for stroke and systemic embolism cannot be established from these findings. Prospective validation against thromboembolic events, in comparison with CHA_2_DS_2_-VASc, is required. Sixth, the framework addresses only the hemodynamic component of Virchow's triad, without incorporating coagulation factors, blood composition, or tissue-level properties. Clinical translation toward stroke prediction would require integration with coagulation biomarkers, clinical risk scores, and tissue characterization methods. Finally, the CFD analysis relied on CT-derived anatomical models, and radiation exposure limits were applicable for serial monitoring. Comparative studies with 4D flow MRI would clarify cross-platform feasibility.

## 5 Conclusion

This study demonstrated that radiomics analysis of CFD-derived hemodynamic maps can quantify left atrial flow patterns in AF. The classifier achieved 93% accuracy, 95% sensitivity, and 90% specificity (AUC = 0.92) in distinguishing AF patients from controls. Permutation testing confirmed that performance significantly exceeded the chance levels (*p* = 0.002).

Five robust radiomics biomarkers were identified through nested cross-validation. Four features measured spatial heterogeneity of throughflow patterns (Gray-Level Non-Uniformity). One feature captured vortical flow complexity in the LAA (Dependence Entropy). These features demonstrated significant correlations with established clinical markers of AF severity, including LA enlargement and MR grade.

In conclusion, this work established a framework for quantitative hemodynamic phenotyping that extends beyond traditional scalar metrics. By capturing spatial complexity of flow fields, this approach offers potential applications in AF risk stratification, treatment selection, and monitoring of atrial remodeling.

Future investigations should prioritize: 1) prospective external validation in independent, multi-center cohorts to confirm generalizability of the identified biomarkers, 2) assessment of temporal robustness by extracting radiomics features across multiple cardiac phases, 3) correlation with clinical outcomes such as stroke and systemic embolism, 4) integration with coagulation biomarkers and clinical risk scores for comprehensive thromboembolic risk modeling, 5) extension to AF subtype discrimination in adequately powered cohorts, and 6) comparison with 4D flow MRI-derived radiomics to enable radiation-free assessment.

## Data Availability

The raw CT data cannot be made publicly available due to privacy concerns and to institutional and national privacy regulations that prohibit public release. The input data used directly in the present analysis will be made available by the authors upon reasonable request.

## Footnotes

^1^ https://pyradiomics.readthedocs.io/en/latest/
