# Supplementary material for: CFD-derived radiomics from hemodynamic maps for quantitative assessment of left atrial flow in atrial fibrillation: a proof-of-concept study
Source: Front Cardiovasc Med. 2026 Jun 4;13:1787926. doi: 10.3389/fcvm.2026.1787926 (PMC13275273; doi:10.3389/fcvm.2026.1787926)
Supplement: Supplementary file 1 [file Datasheet1.pdf]

Supplementary Material

CFD-Derived Radiomics from Hemodynamic Maps for Quantitative Assessment of Left Atrial Flow in Atrial Fibrillation: A Proof-of-Concept Study

Supplementary Material A: Computational Fluid Dynamics Specification

The CFD model applied in this work has been previously published by Masci et al. and validated on controls and AF patients by Falanga et al. (Masci et al., 2020; Falanga et al., 2024). In the following sections, the workflow (Figure A) for CFD simulations is summarized.

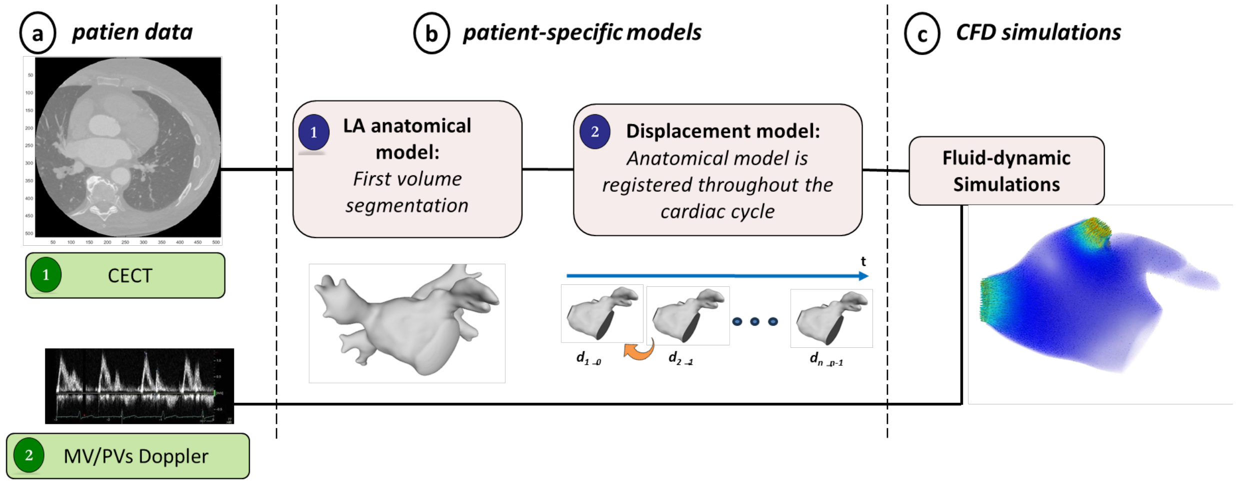

Figure A: Graphical description of the developed CFD workflow: the first step was the acquisition of the contrast enhanced computed tomography (CECT) data and the mitral valve/pulmonary veins (MV/PVs) Doppler data (a); starting from the dynamic CT acquisition, volumes were reconstructed through ECG-gating technique and then the first volume was segmented to define the anatomical model (b1); then, by registering the acquired CT volumes in time, the displacement model was obtained throughout the cardiac cycle (b2). Finally, the displacement model and Doppler data were provided as inputs to the CFD model to perform fluid-dynamic simulations (c).

A.1 Patient-specific LA model

For each CFD simulation, the computational domain was represented by the patient-specific moving LA chamber derived from CT imaging. The 3D LA anatomy, including the four pulmonary veins (PVs) and the left atrial appendage (LAA), was obtained from the ventricular end-diastole CT volume. To provide a smooth geometrical representation of the computational domain, the 3D anatomical model was further filtered by applying Laplacian smoothing using MeshLab software. Five cut planes were applied to the four PVs and the mitral valve (MV) to define inflow and outflow boundary subsets. The final anatomical model was used as input for labeling and volume tetrahedral mesh generation using the VMTK library. The computational domain was discretized using not adaptive tetrahedral elements generated with the VMTK library. The average total element count was 1,138,434 tetrahedra (range: 572,816–2,733,416), corresponding to a mesh density of 14,246 tetrahedra per mL (range: 7,041–36,699).

A.2 Patient-Specific LA Wall Displacement

Patient-specific LA wall displacement throughout the cardiac cycle was computed by image registration of the full CT time sequence using a 3D non-rigid registration based on a B-spline transformation model (Klein et al., 2010), with mean squared difference as the similarity measure. The resulting displacement field was applied to the patient-specific LA model derived from CT imaging. Continuous interpolation of discrete mesh node positions was performed to increase the temporal resolution of the model, ensuring numerical stability during CFD simulation. Fourier series interpolation was applied to achieve a model that accurately represents both the cardiac anatomy and the cyclical nature of its motion.

A.3 CFD Model and Boundary Conditions

The CFD model solved the incompressible Navier–Stokes equations in the Arbitrary Lagrangian–Eulerian (ALE) frame of reference to account for the motion of the computational domain. The Reynolds number in the LA was estimated to be in the range 100–1000, supporting the assumption of laminar flow. Spatial discretization employed the Finite Element method with scalar Lagrangian basis functions of polynomial degree r ≥ 1, with a suitable stabilization scheme (Forti and Dedè, 2015; Reymond et al., 2013). Temporal discretization used a second-order semi-implicit backward differentiation formula (BDF2) scheme (Forti and Dedè, 2015).

PW Doppler velocity measurements at the MV and the PVs acquired before the ablation procedure were used to set realistic boundary conditions for the CFD model (Masci et al., 2020) (Figure A-a2). To compute the MV flowrate, we multiplied the intracardiac MV Doppler velocity by the MV cross-sectional area. The same procedure was applied to compute each PV flowrates. The flowrate Q assigned at each PV was obtained by mass balance taking into account also the flux associated with LA volume variation throughout the cardiac cycle ($Q^{wall} = \frac{dV}{dt}$):

$$Q_1^{pv} + Q_2^{pv} + Q_3^{pv} + Q_4^{pv} + Q^0 + Q^{wall} = 0$$

where Q_l^{pv}, l = {1,2,3,4} are the flowrates at each PV and $Q^0$is the desired MV flow rate.

By setting $Q_1^{pv} + Q_2^{pv} + Q_3^{pv} + Q_4^{pv}$= Q_{TOTAL}^{PV} we derived $Q_{TOTAL}^{PV} = -(Q^o + Q^{wall}\ Q_{TOTAL}^{PV})$ was then distributed among PVs with a criterion based on their sectional areas that moves and changes in shape throughout the cardiac cycle:

$$Q_l^{pv} = \frac{A_l}{A_t} * Q_{TOTAL}^{PV} \text{-}\ Q_l^{w}$$

where Q_l^{pv} , l = {1,2,3,4} is the PVs flow; A_l, l = {1,2,3,4} is the sectional area of each PV; $A_t$is the sum of PVs sectional areas and Q_l^{w}, l = {1,2,3,4} is the flow due to the mesh velocity for each PV. Furthermore, at each PV we imposed a parabolic velocity profile. Regarding the outflow, i.e. MV, we imposed a natural-type boundary condition to penalize backflow (Bazilevs et al., 2009) and avoid unrealistic backflows. The time step was set to 0.001 s. All simulations were performed using the LifeX finite element library (Africa, 2022).

A.4 Solution Convergence and Verification

Convergence at each time step was achieved using a Newton iterative scheme with absolute and relative residual tolerances of 10^{-8}. The linear solver (GMRES) employed a tolerance of 10^{-10}, ensuring numerically converged solutions at every time step. To remove the influence of non-physiological initial conditions on the fluid velocity, 6–7 cardiac cycles were simulated per patient, following (Menghini et al., 2017). The results of the first two or three cycles were discarded. The remaining cycles were phase-averaged: the velocity field at every point of the domain at a given time of the first useful heartbeat was averaged with the corresponding fields at the same point but at corresponding times of subsequent heartbeats. In this manner, a representative average heartbeat was obtained to account for cycle-to-cycle variability due to nonlinearity in the Navier–Stokes equations. Although a true steady state is not expected given the intrinsic nature of the problem, periodic convergence of the solution was achieved.

References for Supplement A

Africa, P.C., 2022. lifex: A flexible, high performance library for the numerical solution of complex finite element problems. SoftwareX 20, 101252. https://doi.org/10.1016/j.softx.2022.101252

Bazilevs, Y., Gohean, J.R., Hughes, T.J.R., Moser, R.D., Zhang, Y., 2009. Patient-specific isogeometric fluid–structure interaction analysis of thoracic aortic blood flow due to implantation of the Jarvik 2000 left ventricular assist device. Comput. Methods Appl. Mech. Eng. 198, 3534–3550. https://doi.org/10.1016/j.cma.2009.04.015

Falanga, M., Cortesi, C., Chiaravalloti, A., Monte, A.D., Tomasi, C., Corsi, C., 2024. A digital twin approach for stroke risk assessment in Atrial Fibrillation Patients. Heliyon 10, e39527. https://doi.org/10.1016/j.heliyon.2024.e39527

Forti, D., Dedè, L., 2015. Semi-implicit BDF time discretization of the Navier–Stokes equations with VMS-LES modeling in a High Performance Computing framework. Comput. Fluids 117, 168–182. https://doi.org/10.1016/j.compfluid.2015.05.011

Klein, S., Staring, M., Murphy, K., Viergever, M.A., Pluim, J.P.W., 2010. elastix: a toolbox for intensity-based medical image registration. IEEE Trans. Med. Imaging 29, 196–205. https://doi.org/10.1109/TMI.2009.2035616

Masci, A., Alessandrini, M., Forti, D., Menghini, F., Dedé, L., Tomasi, C., Quarteroni, A., Corsi, C., 2020. A Proof of Concept for Computational Fluid Dynamic Analysis of the Left Atrium in Atrial Fibrillation on a Patient-Specific Basis. J. Biomech. Eng. 142, 011002. https://doi.org/10.1115/1.4044583

Menghini, F., Dede, L., Forti, D., Quarteroni, A., n.d. Hemodynamics in a left atrium based on a Variational Multiscale-LES numerical model.

Reymond, P., Crosetto, P., Deparis, S., Quarteroni, A., Stergiopulos, N., 2013. Physiological simulation of blood flow in the aorta: comparison of hemodynamic indices as predicted by 3-D FSI, 3-D rigid wall and 1-D models. Med. Eng. Phys. 35, 784–791. https://doi.org/10.1016/j.medengphy.2012.08.009.

Supplementary Material B: Hemodynamic Parameter Definitions

Five hemodynamic parameter maps were computed for each of the eight anatomical planes using *ParaView*. Each parameter captures a distinct aspect of flow orientation, rotation, or helical motion within the LA, providing complementary insights into the underlying hemodynamic behavior (22). For each voxel i on plane p, with local velocity vector ($\vec{v_i}$) (m/s) and plane normal $\vec{n}$, the parameters are defined as follows.

B.1 Throughflow: The Throughflow parameter (u_i) (m/s) quantifies the component of blood velocity perpendicular to a defined anatomical plane, providing a direct measure of net through-plane transport between cardiac regions. It is determined as the scalar product of the local velocity vector and the plane normal:

$$u_i = \vec{v_i} \cdot \vec{n}$$

The resulting values retain the units of velocity (m/s), with positive and negative signs denoting flow direction relative to the plane's normal orientation. Throughflow maps allow visualization of regions where flow enters or exits specific chambers, such as the pulmonary vein inflow or mitral valve outflow planes of the LA. By quantifying through-plane velocity distribution, Throughflow serves as a fundamental descriptor of localized transport efficiency and can highlight altered inflow and outflow organization in AF (22).

B.2 Wall-Parallelity Degree (WPD): The Wall-Parallelity Degree quantifies the directional relationship between local blood flow and the orientation of a reference plane. It is calculated as the ratio between the magnitude of the Throughflow component (u_i) and the total velocity magnitude ($|\vec{v_i}|$):

$$WPD_i = \frac{u_i}{|\vec{v_i}|}$$

WPD values close to 1 indicate that flow is predominantly perpendicular (normal) to the plane's surface, while values near 0 correspond to flow that is largely tangential (parallel) to it. This dimensionless metric (range [0, 1]) captures the orientation and spatial organization of blood flow relative to anatomical structures. Regions with high WPD reflect strong Throughflow indicating efficient inflow or outflow, whereas low WPD values are associated with recirculating or wall-parallel motion, often linked to stagnation zones (22).

B.3 Normalized Vorticity Component (Ω_n): Vorticity ($\vec{\omega_i}$) is defined as the curl of the velocity vector field (1/s), representing the local rotational motion of blood flow. It indicates how rapidly and around which axis fluid elements rotate, providing insight into the formation and strength of vortical structures within the LA.

$$\vec{\omega_i} = \nabla_i \times \vec{v_i}$$

The normalized vorticity component (Ω_n) describes how the direction of local rotational motion is oriented relative to the normal of a selected plane:

$$\Omega_n = \frac{\vec{\omega_i} \cdot \vec{n}}{|\vec{\omega_i}|}$$

This dimensionless measure (range [−1, +1]) indicates whether the local rotational motion is aligned with, perpendicular to, or opposite to the plane orientation. Altered vorticity alignment and reduced coherence of vortical structures are associated with flow disturbances in pathological conditions such as AF (22).

B.4 Local Normalized Helicity (LNH): The Local Normalized Helicity (LNH) is derived from the velocity and vorticity fields, computed as the normalized scalar product of the vorticity vector and the velocity vector:

$$LNH_i = \frac{\vec{\omega_i} \cdot \vec{v_i}}{|\vec{\omega_i}|\,|\vec{v_i}|}$$

LNH provides a dimensionless quantification (range [−1, 1]) of the directional relationship between the velocity and vorticity fields, expressing how closely the local flow direction aligns with the axis of rotation. High positive values (+1) indicate right-handed helical flow; negative values (−1) indicate left-handed helical motion; values near zero describe regions where velocity and vorticity are nearly orthogonal, reflecting weak or disorganized rotational flow. Physiologically, helicity reflects the degree of flow organization and energy efficiency within a chamber. A decrease in LNH magnitude is typically associated with flow instability, energy dissipation, or loss of coherent rotational motion (22).

B.5 Flow Angle (α_i): The Flow Angle describes the geometric orientation of blood flow relative to an anatomical plane. It is defined as the angle between the local velocity vector and the plane normal, computed as the inverse cosine of the normalized scalar product:

$$\alpha_i = cos^{-1}(\frac{\vec{v_i}}{|\vec{v_i}|} \cdot \vec{n})$$

The angle is expressed in degrees (0°–180°), where smaller angles indicate flow nearly perpendicular to the plane (direct through-plane passage) and larger angles represent more tangential or parallel flow. From the LA flow perspective, (α_i) reflects the degree of alignment between regional blood motion and anatomical pathways, offering a complementary perspective to Throughflow for identifying regions of disturbed or redirected flow patterns (22).

Supplementary Material C: Hemodynamic Parameter Map Normalization

All hemodynamic parameter maps were normalized using deterministic, fixed-range rescaling applied identically to every subject. The normalization mappings, defined *a priori* based on the actual numerical ranges computed from the raw CFD output maps across all subjects, are specified in Table C. The rescaled output ranges follow the conventions established by Huellebrand et al. (2023) for radiomics analysis of hemodynamic parameter maps. Because the input and output ranges are fixed constants applied identically to every subject (not derived from per-subject statistics such as percentiles or min/max), the normalization is fully deterministic and introduces no subject-dependent bias.

Table C. Deterministic normalization ranges for hemodynamic parameter maps.

Parameter	Input Range	Output Range	Symmetry	Ref.
Throughflow	[−2, 2] m/s	[−2048, 2047]	Symmetric	(22)
WPD	[0, 1]	[0, 4096]	Unsigned	(22)
Flow Angle	[0°, 180°]	[0, 4096]	Unsigned	(22)
LNH	[−1, 1]	[−2048, 2047]	Symmetric	(22)
Ω_n (Vorticity)	[−1, 1] 1/s	[−2048, 2047]	Symmetric	(22)

Output ranges provide 4096 discrete intensity levels (12-bit depth) for texture feature computation, following the convention of Huellebrand et al. (22). WPD: Wall-Parallelity Degree; LNH: Local Normalized Helicity; Ω_n: Normalized Vorticity component.

Supplementary Material D: Complete Radiomics Pipeline.

The radiomics pipeline comprised deterministic preprocessing applied identically to all subjects, followed by feature extraction. The details are described in the following sections.

D.1 VTI-to-NRRD Conversion

Hemodynamic parameter maps exported from ParaView as VTI (VTK ImageData) files were converted to NRRD (Nearly Raw Raster Data) format for compatibility with PyRadiomics. The conversion preserved spatial geometry (origin, spacing, dimensions) and scalar intensity values. For each subject, 40 NRRD files were generated (5 parameters × 8 planes). All maps maintained 512 × 512 in-plane resolution with 1-slice thickness.

D.2 Binary Mask Generation

Binary ROI masks were derived from the LNH parameter map for each plane using a deterministic threshold: voxels with $|LNH| > \varepsilon$ were assigned label 1 (foreground), where $\varepsilon = \max(10^{-12}, 10^{-4} \times \text{range})$. This threshold isolates flow-relevant regions while excluding zero-valued background voxels. The same mask was applied across all five parameter maps for a given plane, ensuring spatial consistency. All masks were visually verified in 3D Slicer.

D.3 Spatial Resampling

All parameter maps were resampled to isotropic 1 × 1 mm^2 in-plane resolution using linear interpolation via SimpleITK. The thin axis (slice direction) was preserved unchanged. Masks were resampled to the same grid using nearest-neighbor interpolation to avoid label interpolation artifacts. The target spacing (0.001 m = 1 mm) was a fixed constant applied identically to all subjects.

D.4 Extracted Feature Classes

Features were extracted using PyRadiomics version 3.0.1 in accordance with IBSI guidelines. For each plane–parameter combination, 109 features were extracted per subject, grouped into the following IBSI-compliant classes (Table D).

Table D. Radiomics feature classes and counts per plane–parameter pair.

Feature Class	Abbreviation	No. Features
First-Order Statistics	—	18
2D Shape Descriptors	Shape2D	10
Gray-Level Co-occurrence Matrix	GLCM	24
Gray-Level Run Length Matrix	GLRLM	16
Gray-Level Size Zone Matrix	GLSZM	16
Gray-Level Dependence Matrix	GLDM	14
Neighboring Gray Tone Difference Matrix	NGTDM	5
**Total per plane–parameter pair**		**109***

*Note: The manuscript reports 360 features because only features with non-zero variance across subjects were retained (approximately 9 features per plane–parameter pair, × 8 planes × 5 parameters = 360 after QC filtering of near-constant features). Features with standard deviation < 10^{-10} were excluded.

Supplementary Material E: Classification Pipeline Configuration

The complete machine learning pipeline parameters are specified in Table E.

Table E. Machine learning pipeline parameters.

Parameter	Value	Rationale
Classifier	SVM (RBF kernel)	Effective for small-sample radiomics
C (regularization)	1.0	Default; no hyperparameter tuning
Gamma	'scale' (1 / (n_features × var))	Automatic scaling
Class weights	Balanced (inversely proportional)	Address 10:20 class imbalance
Feature standardization	Z-score (per fold)	Prevent data leakage
Validation	Nested LOOCV (n = 30)	Maximize data utilization
Random seed	123	Reproducibility
Max features per fold	5 (n/p = 6:1)	Overfitting mitigation
Bootstrap iterations	1000	Stability assessment
Bootstrap sampling fraction	70% per group (stratified)	Stability selection theory
Permutation iterations	1000	Chance-level testing
FDR threshold (α)	0.10	Benjamini–Hochberg
Effect size threshold (ε^2)	≥ 0.14	Large effect (non-parametric)
Stability threshold	≥ 70%	Meinshausen & Bühlmann (2010)

Software: Python 3.10, PyRadiomics 3.0.1, scikit-learn 1.3, SimpleITK, SciPy 1.11, NumPy 1.26, pandas 2.1, SHAP 0.43, Matplotlib 3.7, Seaborn.

Confidence intervals: Bootstrap resampling (1000 iterations, percentile method) was used to compute 95% confidence intervals for all metrics: accuracy 93.3% (95% CI: 83.3–100.0%), sensitivity 95.0% (95% CI: 84.0–100.0%), specificity 90.0% (95% CI: 66.7–100.0%), and AUC-ROC 0.92 (95% CI: 0.78–1.00). The relatively wide CIs, particularly for specificity, reflect the small control group (n = 10) and are expected given the sample size. Notably, the lower bound of the AUC CI (0.78) remains well above chance level, consistent with the permutation testing result ($p = 0.002$), shown in Figure E.

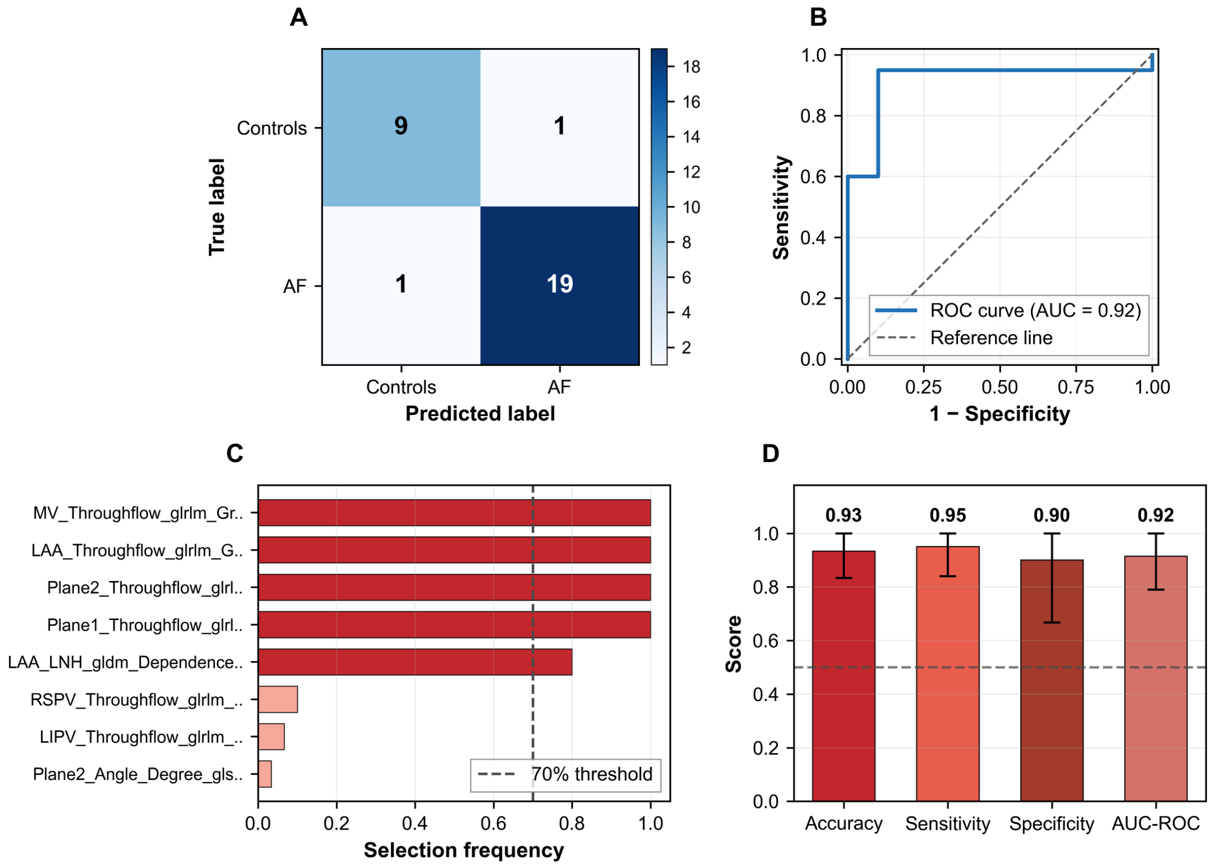

Figure E. SVM classifier performance for AF versus control classification (n = 30). (A) Confusion matrix showing classification outcomes across nested LOOCV folds (19/20 AF and 9/10 controls correctly classified). (B) Receiver operating characteristic curve (AUC = 0.92; 95% CI: 0.78–1.00), with the dashed diagonal representing chance-level classification. (C) Radiomics feature selection frequency across 30 LOOCV folds; the dashed line indicates the 70% stability threshold. (D) Overall classification metrics with 95% bootstrap confidence intervals (1000 iterations); the dashed line indicates the permutation null distribution mean (65.9%, p = 0.002).

Supplementary Material F: Incremental Analysis: Clinical Variables vs. Radiomics Features

To assess whether the radiomics signal reflects AF-specific hemodynamics or associated structural remodeling, an incremental analysis was performed comparing three models using the same nested LOOCV framework (SVM-RBF, C = 1.0, gamma = ‘scale’, balanced class weights). Clinical variables included age, gender, body surface area, left ventricular ejection fraction, LA enlargement grade, and mitral regurgitation grade (6 features). Results are summarized in Table F.

Table F. Incremental analysis comparing clinical-only, radiomics-only, and combined models.

Model	Features	Accuracy	Sensitivity	Specificity	Bal. Acc.	AUC-ROC
Clinical variables only	6	63.3%	60.0%	70.0%	65.0%	0.57
**Radiomics features only**	**360**	**93.3%**	**95.0%**	**90.0%**	**92.5%**	**0.92**
Clinical + Radiomics	366	93.3%	95.0%	90.0%	92.5%	0.92

Clinical variables: age, gender, BSA, LV EF, LA enlargement grade, MR grade. All models used the same nested LOOCV framework with SVM-RBF classifier (C = 1.0, gamma = ‘scale’, balanced class weights). Bold values indicate best performance. The clinical-only model performed near chance level (AUC = 0.565), while radiomics features achieved AUC = 0.915. Adding clinical variables to radiomics produced no incremental improvement, confirming that hemodynamic texture features capture spatial flow complexity beyond what clinical surrogates of atrial remodeling provide.
